# Isolation and Identification of Bioactive Compounds from *Bidens* spp. Using HPLC-DAD and GC-MS Analysis and Their Biological Activity as Anticancer Molecules

**DOI:** 10.3390/molecules27061927

**Published:** 2022-03-16

**Authors:** Kulsoom Zahara, Yamin Bibi, Saadia Masood, Sobia Nisa, Ahmad Sher, Naushad Ali, Sunjeet Kumar, Abdul Qayyum, Waseem Ahmed, Rokayya Sami, Amina A. M. Al-Mushhin, Amani H. Aljahani

**Affiliations:** 1 Department of Botany, PMAS-Arid Agriculture University Rawalpindi, Rawalpindi 46300, Pakistan; kulsoomzahara@gmail.com; 2 Department of Statistics & Mathematics, PMAS-Arid Agriculture University Rawalpindi, Rawalpindi 46300, Pakistan; saadia.masood@uaar.edu.pk; 3 Department of Microbiology, The University of Haripur, Haripur 22620, Pakistan; sobia@uoh.edu.pk; 4 College of Agriculture, Bahauddin Zakariya University, Bahadur Sub Campus, Layyah 31200, Pakistan; ahmad.sher@bzu.edu.pk; 5 Department of Plant Breeding & Genetics, The University of Haripur, Haripur 22620, Pakistan; naushadali@uoh.edu.pk; 6 Key Laboratory for Quality Regulation of Tropical Horticultural Crops of Hainan Province, School of Horticulture, Hainan University, Haikou 570228, China; 184224@hainanu.edu.cn; 7 Department of Agronomy, The University of Haripur, Haripur 22620, Pakistan; 8 Department of Horticulture, The University of Haripur, Haripur 22620, Pakistan; dr.waseemahmed@uoh.edu.pk; 9 Department of Food Science and Nutrition, College of Sciences, Taif University, P.O. Box 11099, Taif 21944, Saudi Arabia; rokayya.d@tu.edu.sa; 10 Department of Biology, College of Science and Humanities in Al-Kharj, Prince Sattam Bin Abdulaziz University, Al-Kharj 11942, Saudi Arabia; a.almushhin@psau.edu.sa; 11 Department of Physical Sport Science, College of Education, Princess Nourah bint Abdulrahman University, P.O. Box 84428, Riyadh 11671, Saudi Arabia; ahaljahani@pnu.edu.sa

**Keywords:** anticancer, *Bidens*, zebrafish, brine shrimp

## Abstract

The genus *Bidens* a member of family Compositae, is widely documented as an ethno-medicinally important genus of plants. In the present study, anticancer potential of three ethno-medicinally important species i.e., *B. bipinnata*, *B. biternata* and *B. pilosa* were tested. For in-vitro evaluation, an MTT (Thiazolyl blue tetrazolium bromide) assay was performed against cervical cancer cells (HeLa), hepatocellular carcinoma (HepG), and adenocarcinoma human alveolar basal epithelial cells (A549). For in vivo evaluation, *Artemia salina*, *Danio rerio,* and *Caenorhabditis elegans* were used. Among all the tested extracts, the ethanol extract of *B. biternata* appeared to have highest anticancer activity, and the compounds responsible for this activity were identified to be Tris (2,4-di-*tert*-butylphenyl), 4-hydroxy-2,4′-dimethoxychalcone, and 2,4-di-*tert*-butylphenol. This is the first report of the isolation of Tris (2,4-di-*tert*-butylphenyl) phosphate from the genus *Bidens* and the first report of 4-hydroxy-2,4′-dimethoxychalcone and 2,4-di-*tert*-butylphenol from *B. biternata*. Among the isolated compounds, 4-hydroxy-2,4′-dimethoxychalcone showed the highest anticancer activity with an LD50 value of 236.7 µg/mL. Therefore, this compound carries promising potential for being established as a pharmaceutical for chemoprevention and chemotherapy.

## 1. Introduction

Overproduction of several reactive oxygen species, i.e., oxygen radicals and non-free radical species is reflected to be the chief provider to oxidative stress, which has been associated to numerous ailments like cancer, tissue damage in rheumatoid arthritis and atherosclerosis [1]. Plants are a representative source of drugs and edible plant are chief sources of antioxidants that have the capability to defend the body from injury produced by free radicals prompted oxidative stress [2].

The protective mechanisms of phytochemicals on tumor advancement vary from the inhibition of genotoxic effects to inhibition of proteases and cell proliferation, better antioxidant activity, signal transduction pathways and defense of intracellular infrastructures to control apoptosis [3].

*Bidens* is a widespread genus consisting of 247 species that are cosmopolitan in distribution [4]. Many of these species have been reported to have sesquiterpene, acetylenes, lactones and flavonoids [5]. However, the most widespread are aromatic derivatives, thiophenes, carotene, coumarins (umbelliferon, scopoletin and aesculetin), vitamin C and C17-, C14-, C13-polyacetylenes [6]. *B. pilosa* is reported to cure various ailments i.e., infectious diseases, immunological disorders, metabolic syndrome and etc. [7]. This herb is taken in the form of decoction, infusion or juice. However, in the case of snakebite and bleeding wounds it can be applied externally (Table 1). It can be used alone or with other medicinal plants i.e., *Cissus sicyoides, Aloe vera, Valeriana officinalis* and *Plectranthus mollis* [8].

*B. bipinnata* is emmenagogue, stimulant, antispasmodic, and have expectorant effect. Traditionally it is used to treat laryngeal, asthma and respiratory disorders (Table 1). In vivo studies of *B. bipinnata* extract has shown antimalarial effect. Its ethanol extract shows 70% inhibition of plasmodium growth [9]. The studies have reported that butanol extract of *B. biternata* have showed a very high antiradical potential whereas its n-hexane extract showed very low antiradical potential [10]. The main constituents of *B. biternata* are saponins, steroids, terpenoids, coumarins, glycosides, athraquinones, iridoids, alkaloids, tannins, phlobatannins and flavonoids [10]. In the present study three species of genus Bidens has been investigated for their anticancer potential using multiple in-vivo and in-vitro assays and the compounds responsible for these activities were isolated and analyzed for their subsequent activity.

**Table 1:** Ethno-medical evidence about *B. pilosa*, *B. biternata,* and *B. bipinnata*.

Disorder	Plant Part	Dosage Form	Region/Country	References
*B. pilosa*				
Stomach ache	LE	Not stated	Africa	[11]
Colic	WP	Decoction	China, Africa	[11]
Catarrh	WP	Juice	Cuba	[12]
Diarrhea	LE, WP	Decoction	Uganda, Africa	[13]
Constipation	WP	Decoction	India	[14]
Dysentery	WP	Infusion	Africa	
Choleretic	WP	Decoction	America	[15]
Antirheumatic	RT, WP	Infusion	Hong Kong	[16]
Appendicitis	WP	Not stated	Hong Kong	[16]
Enteritis	WP	Decoction	China	[17]
Otitis	WP	Decoction	China, Africa	[18]
Gastritis	WP	Juice	Cuba	[19]
Diabetes	WP	Decoction	Taiwan, Cuba	[11]
Headache	WP	Decoction	Bafia, Cameroon	[11]
Diuretic	WP	Decoction	Central America	[20]
Hypotensive	WP	Juice	Cameroon	[21]
Fever	WP	Decoction	Not stated	[19]
Yellow Fever	LE, WP	Decoction	America	[13]
Acute hepatitis	WP	Decoction	Hong Kong	[22]
Intestinal worms	LE	Decoction	Africa	[14]
Malaria	WP	Juice	China	[18]
Eye diseases	LE	Juice	Uganda	[13]
*B. biternata*				
Leprosy	LE	Not stated	India	[23]
Cuts and wounds	LE	Decoction	India	[23]
Nose bleeds	WP	Decoction	China	[23]
Gastric ulcers	LE	Maceration taken orally	Central America	[10]
Skin problems	WP	Topical application	Africa	[24]
Wounds	WP	Crushed herb	China	[24]
Snake bites	WP	Crushed herb	China	[23]
*B. bipinnata* L.				
Asthma	WP	Decoction	China	[25]
Colds	LE	Decoction	China	[25]
Fever	WP	Decoction	Not stated	[9]
Antimicrobial	AP	Decoction	Trinidad	[26]
Eye Diseases	LE	Juice	Uganda	[13]
Colds	LE, WP	Decoction	Uganda, China	[21]

LE: leaves; WP: whole plant; AP: aerial parts; RT: root.

## 2. Material and Method

### 2.1. Formation of Crude Methanolic Plant Extract

**Figure 1:**
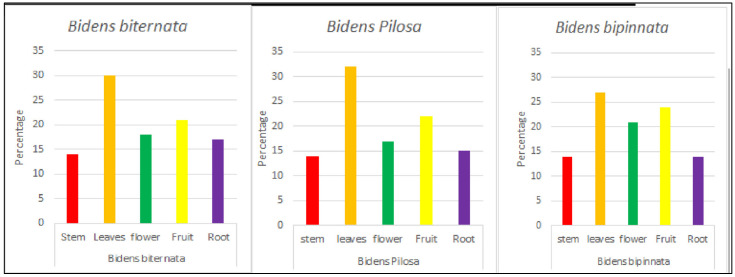
The percentage yield of plants obtained.

The plants parts were collected during October 2016 to October 2017. After collection the collected parts were thoroughly washed, fully desiccated and ground into fine powder. Powdered plant material (150 g) is measured, and using cold maceration technique crude methanol extract is prepared (Figure 1).

The crude extracts obtained were then subjected to liquid-liquid partition. All the solvents used are (HPLC)-grade purity from Sigma-Aldrich Co. (St. Louis, MI, USA). In 250 mL water, extract will be suspended separately and partitioned with n-hexane in a separating funnel. The hexane layer and aqueous layers were collected and concentrated in rotary evaporator. In the concentrated aqueous layer acetone was added and placed in sonicator bath for one hour. The acetone soluble supernatant was separated and dried as acetone extract whereas the precipitates were again treated with ethanol and placed in sonicator bath. The supernatant was concentrated as ethanol extract and precipitates were taken as aqueous extract.

### 2.2. In-Vitro Cytotoxicity Assay

During evaluation of cytotoxicity plant parts i.e., stem, root, leaves, flowers and achenes were separately tested in parallel with four different solvents i.e., ethanol, hexane, acetone, water using MTT assay. In in-vitro conditions three different cell lines i.e., cervical cancer cells (Hela), hepatocellular carcinoma (HEPG) and adenocarcinomic human alveolar basal epithelial cells (A549) were used. This assay is an inexpensive, standard method, to measure cell death. It is established on the reduction of to formazan crystals (purple) due to metabolic active cells [27].

#### Thiazolyl Blue Tetrazolium Bromide (MTT)

Product Number M 2128 (Lab M Ltd., Lancashire, UK); Storage Temperature 2–8 °C.

Procedure

From a cultured plate, cells were dislocated using trypsin (T4799 Sigma-Aldrich (St. Louis, MO 63118, USA). In a flask 5 mL of complete media (D5796 Sigma-Aldrich (Hamburg, Germany)) is added to trypsin zed cells. The trypsinized cells were centrifuged in a 15 mL falcon tube (500 rpm for 5 min). Cell culture media is removed and cells were suspended to 1.0 mL culture media. Suspended cells were counted and by using complete media the cell suspension is diluted (75,000 cells/mL). In a 96 well plate 100 µL of suspended cells were added into each well. The plate is placed in CO_2_ incubator. Next day tested extracts were added and final volume is kept to 100 µL per well. To each well 20 µL of MTT is mixed. As a control wells with no MTT are used. These plates are placed for 3.5 h at 37 °C in CO_2_ incubator. The Absorbance is obtained at 590 nm using a reference filter of 620 nm.

### 2.3. In-Vivo Cytotoxicity Assay Caenorhabditis elegans 

The *C. elegans* (N2 wild-type) were used. At L4 stage the worms are subjected for synchronization. The synchronized populations were acquired using alkaline bleaching method [28]. In a 96-well microplate (10 μL, ∼40–45 L4 synchronized larvae were added with 189 μL of *E. coli* OP50 culture (OD = 0.5 at 620 nm and, 1 μL of plant extract. As a solvent control DMSO (1 μL) was used and Levamisole (final concentration 50 μM) Levamisole (Sigma-Aldrich (St. Louis, MO 63118, USA)). The microplate was incubated for 16 h at 20 °C into a WMicroTracker. The movement was recorded every 30 min by the WMicroTracker.

### 2.4. In Vivo Cytotoxicity Assay on Zebrafish

The in-vivo toxicity test is performed on zebrafish larva using permitted protocol of Institutional Animal Ethics and Biosafety Committee of KU Leuven, Belgium [29]. The toxicity assay was performed in 96-well plate. In each well 199 μL of E3 medium with 3 zebra fish larva is with 1 μL of plant extract transferred. DMSO (1 μL) was used as solvent control and gossypol (1 μL) was used as drug control. The microplate was placed into a WMicroTracker for 48 h at 28 °C. The movement of zebrafishes is measured by the WMicroTracker after every 30 min. The plate was also analyzed with microscope to see the number of deaths.

### 2.5. In Vivo Cytotoxicity Assay on Brine Shrimp

The prepared extracts and fractions were tested against brine shrimp lethality test (BSLT) as described [30]. Brine shrimp (*Artemia salina*) eggs (JBL Artemiopur, Germany) were placed in well aerated artificial sea water. A two chambered container was used with one chambered covered whereas other chamber open. In the middle of the two chambers small openings were present. Sea water was prepared (38 g of sea salt/one liter of distilled water). To feed hatched larvae a pinch of yeast was added. After 24 h, the shrimp’s larvae were ready to be used. 

The tested extracts were dissolved in 100% DMSO (stock). From stock different concentrations of solution were prepared using artificial sea water. As a positive control Nicotine N3876 Sigma-Aldrich was utilized. The phototropic nauplii were collected and 10 nauplii were added in each container. After 24 h incubation at room temperature dead nauplii were counted and percentage lethality was measured according to the following formula:

Percentage of Death = (Total nauplii − Alive nauplii) × 100/Total nauplii

### 2.6. Bioassay-Guided Purification

On silica gel (70–230 mesh) dried plant extract was adsorbed and loaded on silica column (600 mm height × 55 mm diameter). Elution were obtained with an increased polarity gradient of hexane–dichloromethane i.e., 9.5:0.5, 9:1, 8.5:0.5, 8:2, 7:3, 6:4, 5:5, 4:6, 3:7, 2:8, 1:9 and 0:10. After that elutions were collected with 100% dichloromethane, and 100% ethyl acetate, ethyl acetate and methanol (9.5:0.5, 9:1, 8:2, 7:3, 6:4, 5:5, 4:6, 3:7, 2:8, and 1:9) and 100% methanol. 

The entire process was supervised at 280 nm and 254 by a Dual λ absorbance detector (Waters Milford, MA 01757, USA). 5 µL Aliquots of 225 fractions were marked on large TLC glass plates (20 cm × 20 cm) and placed in glass jars (20 cm × 10 cm × 20 cm), with mobile phase at room temperature. The plates were monitored using ultra-violet (UV) light at 254 and 360 nm. 

Using Shimadzu, LC-20AT system equipped with LC-20AT quaternary pump, a on-line degasser, a photodiode array detector HPLC was done. The mobile phase of 30:70 H_2_O and acetonitrile was used. 

### 2.7. Identification of Isolated Compound

Collected peaks were exposed to a gas chromatograph along with a mass spectrometer. A Restek RXi-5sil MS 20 m column was utilized. Helium is injected with a rate of 0.9 mL/min. The temperature was gradually adjusted at 20 °C, 120 °C, 200 °C, 250 °C and to end with to 350 °C for 4 min.

## 3. Results and Discussion

Plants have played a vital role in human healthcare management [31]. Medicinal plants are defined as plants that have healing properties or provide valuable pharmacological effects on the body [32]. 

In the present study four different solvents i.e., hexane, acetone, ethanol and water were used and different plant parts of selected plants of genus *Bidens* i.e., root, stem, leaves, flowers and achenes were tested. Three different cell lines were tested i.e., Hela (cervical cancer), HEPG (liver hepatocellular carcinoma) and A549 (adenocarcinoma human alveolar basal epithelial cells). Our results indicate that the ethanol extracts of all tested plants and their parts were appeared to have cytotoxic activity. Studies conducted by Karagöz [33] and Nemati [34] also indicate that ethanol extracts of plants contain some cytotoxic compounds. It was also observed that root extracts of all tested plants have significant activity against all tested cell lines.

**Figure 2:**
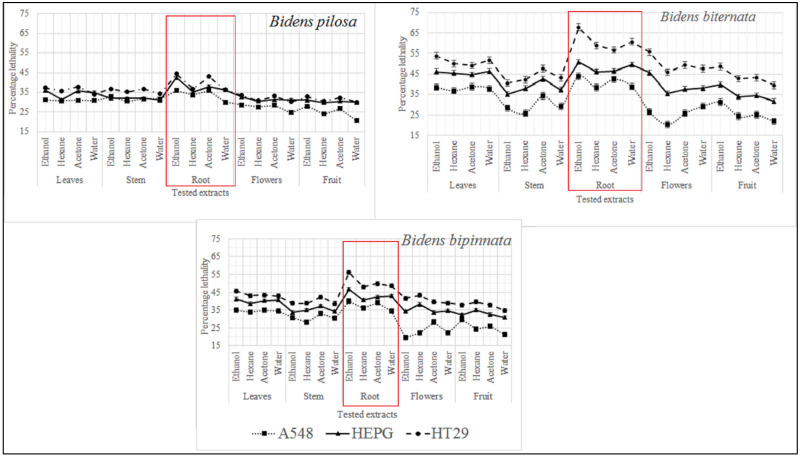
Cytotoxic activity of selected plants of the genus *Bidens* against cervical cancer cells (HeLa), hepatocellular carcinoma (HepG), and adenocarcinoma human alveolar basal epithelial cells (A549).

As described in Figure 2 it was observed that the most active extract is ethanol extract of *B. biternata* with percentage inhibition of 67.75% against HT29, 50.82% against HEPG and 43.8% against A549 cell lines. This is also worth mentioning that roots extract of all the tested plants are appeared to significant activity against all the tested cell lines (Figure 2).

**Figure 3:**
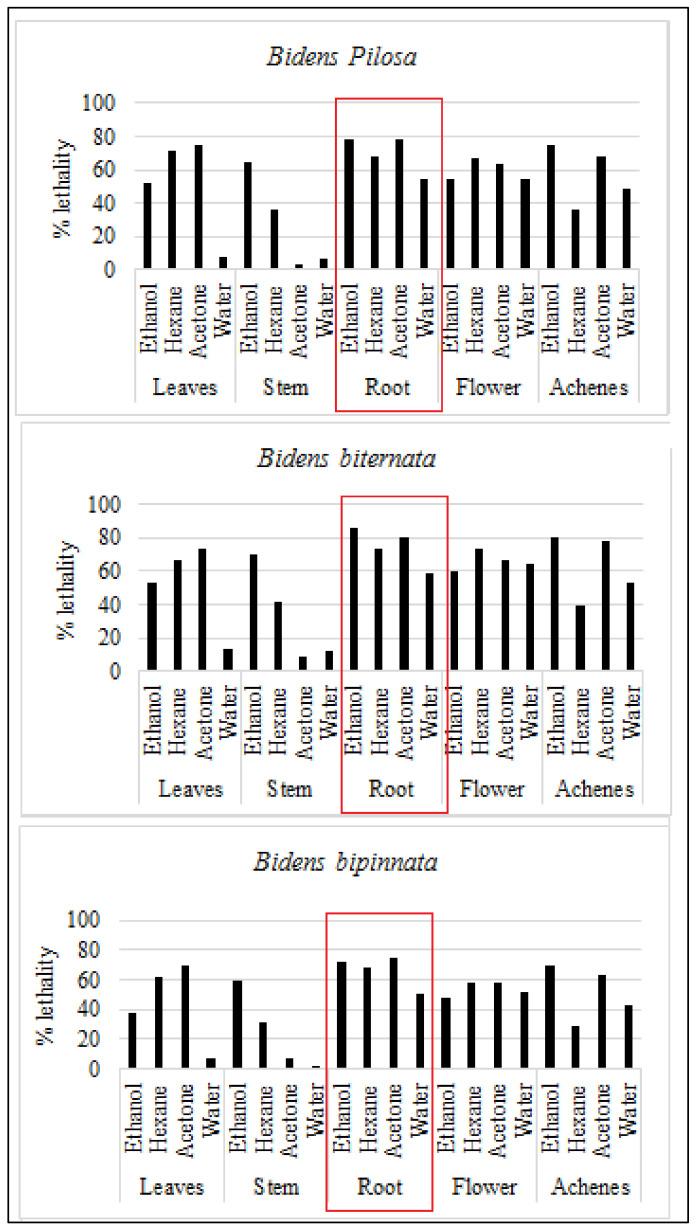
Mean percentage death (lethality) of selected plants against brine shrimp.

For initial toxicity screening brine shrimp lethality bioassay is performed. Brine shrimp assay is appeared to be interrelated with human nasopharyngeal carcinoma cytotoxicity. Our results are in line with previous in-vitro assessment as roots of all the tested plants are appeared to have toxic effect. The ethanol extract of *B. biternata* roots showed highest percentage lethality i.e., 86.67% (Figure 3). 

**Figure 4:**
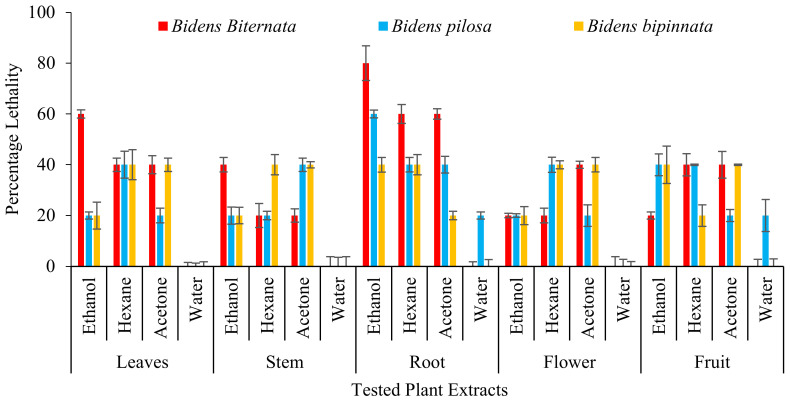
Percentage death of Zebra fishes after treatment of plant extracts.

Zebra fish is been used as an ideal vertebrate organism used in diverse research zones as ecotoxicology, genetics and developmental biology [35]. Studies have showed that humans show great genetic resemblances of genomic sequences and brain patterning with zebra fish. Therefore, this makes zebra fishes a beneficial assay in exploring many toxicology studies yielding a rapid outcome. On zebra fishes all the extracts of *B. biternata* roots except water extract show toxic effect i.e., 80% (ethanol), 60% (hexane) and 60% acetone (Figure 4). Root ethanol extract of *B. pilosa* is also appeared to have toxic effect with percentage lethality of 60%. It is also worth noting that water extract of all tested plant species have less or no toxic effect on zebra fishes. 

**Figure 5:**
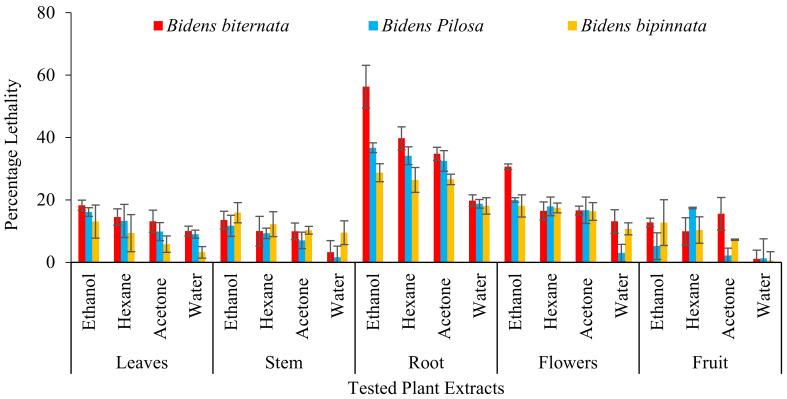
Toxicity assessment on *Caenorhabditis elegans*.

The *Caenorhabditis elegans* a nematode, is remarkably well deliberated animal model and several investigators have utilized it for evaluation of toxicity. They offer a channel among in-vitro tests and mammalian toxicity analysis by merging conventional in-vitro management practices and oral toxicity experiment records from a complete organism [36]. During present analysis on *C. elegans* toxicity it is observed by *B. biternata* root ethanol extract with percentage inhibition of 53.32% as compared to control (Levamisole) i.e., 91.93% (Figure 5).

**Figure 6:**
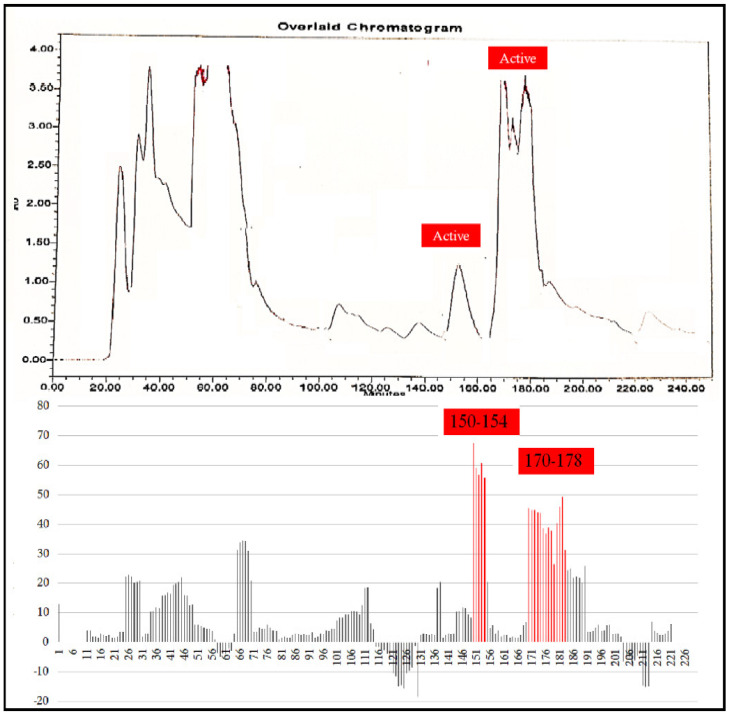
Top panel: Overlaid chromatogram of Ethanol extract of *B. biternata* leaves; percentage inhibition of obtained fractions against cervical cancer cells (Hela) (bottom panel).

**Figure 7:**
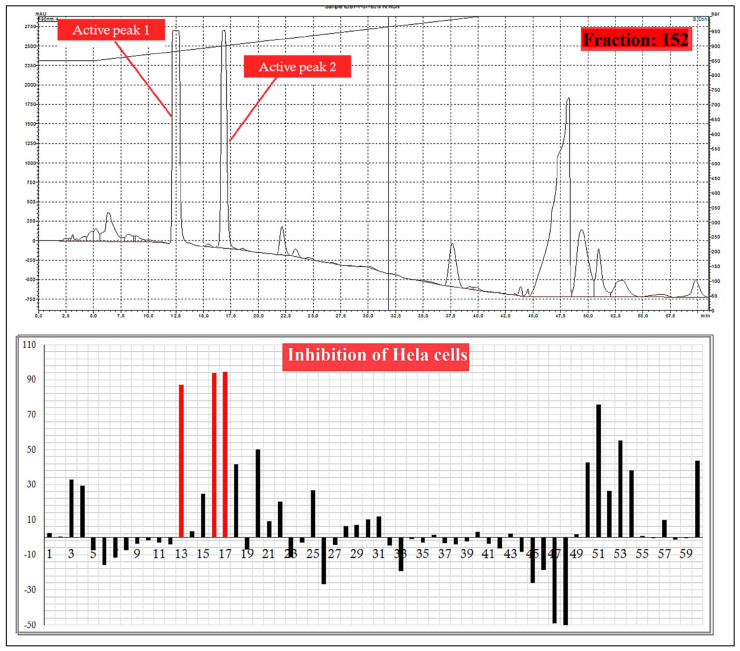
HPLC chromatogram of fraction 152 of silica gel column; percentage inhibition of obtained fractions against cervical cancer cells (Hela) (bottom panel).

**Figure 8:**
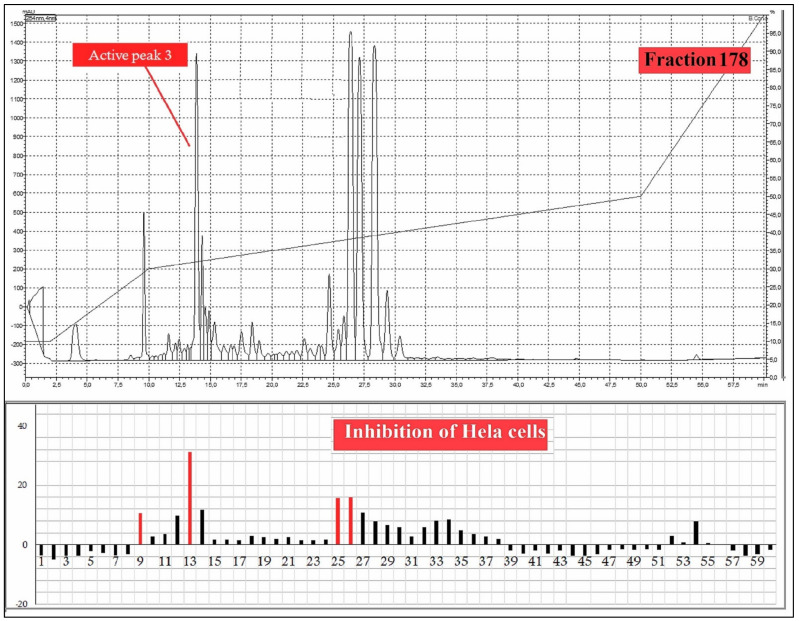
HPLC chromatogram of fraction 178 of silica gel column; percentage inhibition of obtained fractions against cervical cancer cells (Hela) (bottom panel).

A total 225 fractions were collected from silica column chromatography fraction 152 and 178 is appeared to be active against HeLa cell line (Figure 6). These fractions were again using HPLC-DAD analysis (Figure 7 and Figure 8) and active fractions were collected. Before further analysis the purity of collected peaks were tested. Using thin layer chromatography their purity is tested using hexane: ethyl acetate mobile phases. A total of three pure active compounds were identified and were again subjected to gas chromatography mass spectrometry.

**Figure 9:**
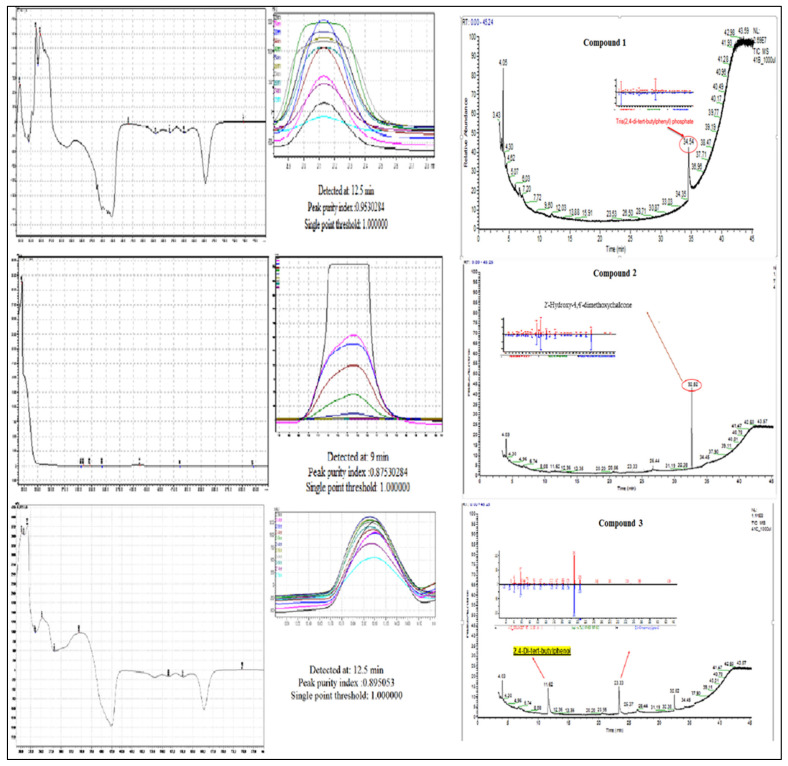
UV–vis apex absorption spectra and mass spectra of Compound **1** Tris (2,4-di-*tert*-butylphenyl), **2** (4-Hydroxy-2,4′-dimethoxychalcone), and **3** (2,4-di-*tert*-butylphenol).

Compound **1** is a white-coloured solid and identified to be Tris (2,4-di-*tert*-butylphenyl) phosphate, C_42_H_63_O_4_P (Figure 8 and Figure 9). Tris (2,4-di-*tert*-butylphenyl) phosphate is an organophosphorus compound which is a phosphate ester derived from di-tart-butylphenol. It has also been identified from the flowers of *Camellia sasanqua* Thunb. [37], *Aquilaria sinensis* (Lour.) Gilg [38] and the leaves of *Chimonanthus* spp. [39]. 3,5-DTBP is reported in the flowers of *Aquilaria sinensis* (Lour.) Gilg [40] and the seeds of *Plukenetia volubilis* L. [41]. From genus *Bidens*, this compound has previously been identified from *B. Pilosa* [42]. However, this compound is not reported in *B. biternata*.

**Figure 10:**
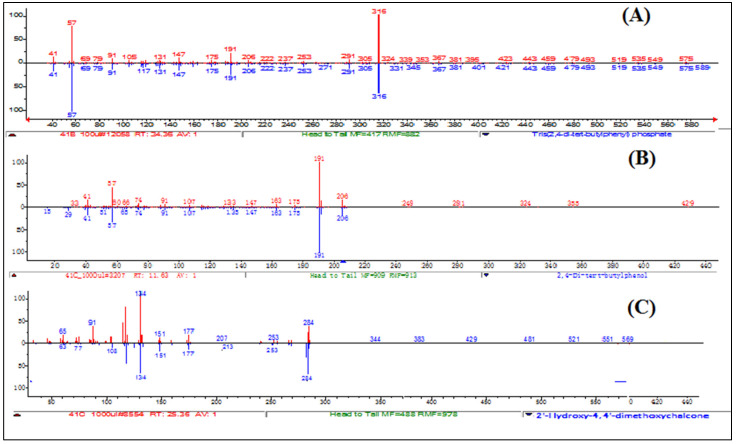
Mass spectra of Compound (**A**) Tris (2,4-di-*tert*-butylphenyl), (**B**) (2,4-di-*tert*-butylphenol), and (**C**) (4-Hydroxy-2,4′-dimethoxychalcone).

Compound **2** is identified to be 4-Hydroxy-2,4′-dimethoxychalcone (Figure 8, Figure 9 and Figure 10). The compound is 4-Hydroxy-2,4′-dimethoxychalcone (C_17_H_16_O_4_) belongs to the class of organic compounds known as chalcones. They are one of the leading classes of flavonoids throughout the entire kingdom of plants. Chalcones are reported to have clinical applications in humans. Previously Licochalcones isolated from licorice has been listed to have an array of biological activities [43].

Compound **3** is a yellow powder identified to be 2,4-di-*tert*-butylphenol (Figure 8, Figure 9 and Figure 10) with molecular formula C_14_H_22_O. This compound is a member of the class of phenols with two *tert*-butyl substituents at positions 2 and 4. This compound is previously been reported from variety of plants i.e., from chloroform and methanol extracts of *Cuscuta reflexa* [44], methanolic extract of *Cordia dicodoma*, *Malvastrum coromandelianum* (L.) Garcke leaves [45]. However, there is no report of this compound “Tris (2,4-di-*tert*-butylphenyl) phosphate” from Asteraceae members.

**Figure 11:**
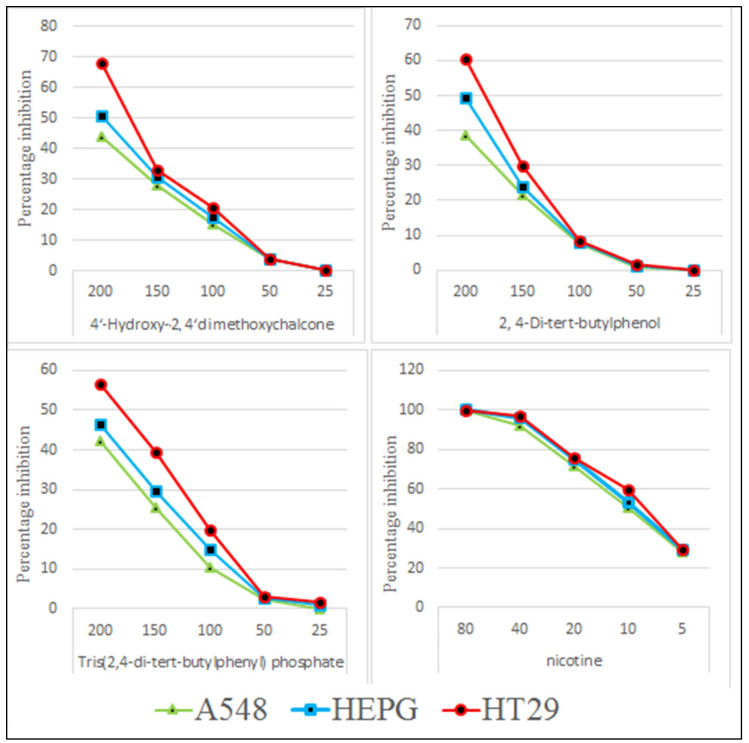
Cytotoxic potential of TDTBP: Tris (2,4-di-*tert*-butylphenyl), HDC: 2 (4-Hydroxy-2,4′-dimethoxychalcone), and DTBP 3 (2,4-di-*tert*-butylphenol).

**Figure 12:**
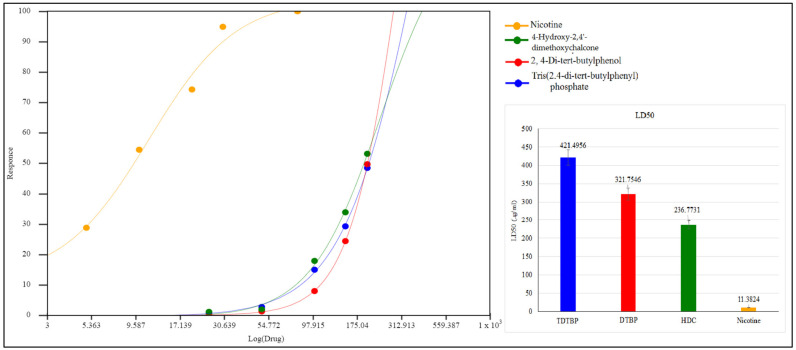
LD50 µg/mL of test compounds TDTBP: Tris (2,4-di-*tert*-butylphenyl), HDC: (4-Hydroxy-2,4′-dimethoxychalcone), and DTBP 3 (2,4-di-*tert*-butylphenol) and the standard (nicotine).

The isolated pure compounds show a moderate cytotoxicity against tested cell lines (Figure 11). Highest activity is observed by 4-Hydroxy-2,4′-dimethoxychalcone with LD50 of 236.7 µg/mL. Previously different reports on cytotoxic activity of chalcones been reported i.e., IC50 values of 45.39 µg/mL and 41.73 µg/mL against MCF-7 and SK-Hep-1 cell lines [46]. 2,4-di-*tert*-butylphenol show a moderate cytotoxicity with LD50 of 321.7 µg/mL (Figure 12). It is proposed that that cytotoxic properties of 2,4-di-*tert*-butylphenol is because it displayed greater results in the initiation of apoptotic [47].

## 4. Conclusions

**Figure 13:**
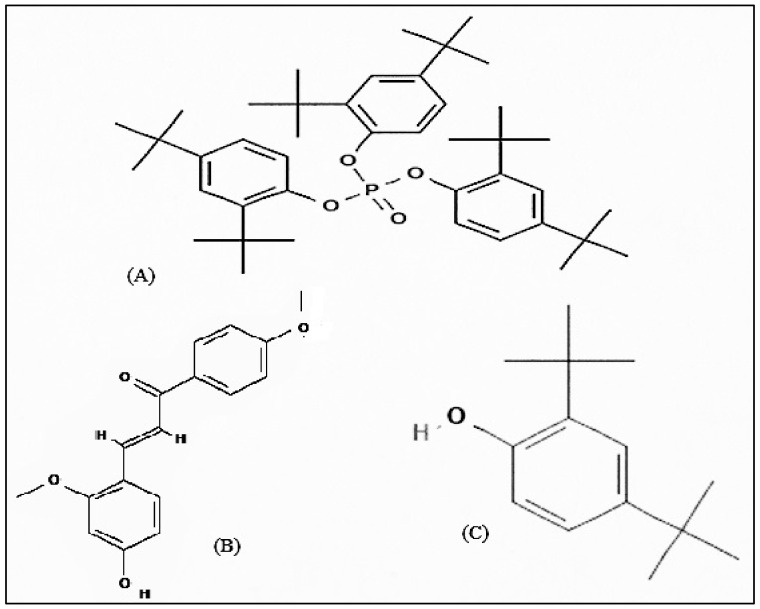
Chemical structure of isolated compounds: (**A**) Tris (2,4-di-*tert*-butylphenyl), (**B**) (4-Hydroxy-2,4′-dimethoxychalcone), and (**C**) (2,4-di-*tert*-butylphenol).

The members of genus *Bidens* are widely documented to be used for treating infectious diseases, immunological disorders, metabolic syndrome, wounds, and many others. In the current study we can conclude that *B. biternata* has anti-cancer constituents active against the Hela, A549 and HEPG cells. The roots ethanol extract of *B. biternata* is appeared to have highest anticancer potential. The compound responsible for anticancer activity are tris (2,4-di-*tert*-butylphenyl), (4-hydroxy-2,4′-dimethoxychalcone) and (2,4-di-*tert*-butylphenol) (Figure 13). These isolated compounds from this extract show a notable anticancer activity especially 4-hydroxy-2,4′-dimethoxychalcone show a promising potential to be chemically standardized for chemoprevention and for treating certain types of cancer in association with conventional treatments.

## Data Availability

Available upon request from the corresponding author.
